# Cardiac warning symptoms in paediatric out-of-hospital ventricular fibrillation cardiac arrest survivors

**DOI:** 10.1136/openhrt-2026-004412

**Published:** 2026-10-05

**Authors:** Jennifer Tollit, Ella Field, Stephanie Oates, Gabrielle Norrish, Elena Cervi, Luke Starling, Juan Pablo Kaski

**Affiliations:** 1 Institute of Cardiovascular Science, University College London, London, UK; 2 Centre for Inherited Cardiovascular Diseases, Great Ormond Street Hospital For Children NHS Foundation Trust, London, UK

**Keywords:** Death, Sudden, Cardiac, Out-of-Hospital Cardiac Arrest, Arrhythmias, Cardiac

## Abstract

**Summary:**

**Background:**

Out-of-hospital cardiac arrest (OHCA) in children is a rare but devastating event, with an estimated incidence of 9 per 100 000 person-years and a survival rate of less than 10%. OHCA may be the presenting event in children with undiagnosed cardiovascular disease. A substantial proportion of adults experiencing an unexpected OHCA report previous symptoms, but whether the same is true in paediatric survivors has not been systematically evaluated previously. This study aims to describe cardiac symptoms in children prior to presenting with OHCA.

**Methods:**

Clinical characteristics and preceding symptoms of 58 consecutive patients aged 0–18 without a pre-existing causative diagnosis presenting with a survived OHCA between 1996 and 2025 to a single UK paediatric tertiary referral centre were reviewed.

**Results:**

The median age at cardiac arrest was 11 years (IQR 7–14 years). 29 OHCA (50%) occurred on exertion. Following the OHCA, 42 patients (72.4%) were diagnosed with an inherited cardiac condition, most commonly hypertrophic cardiomyopathy (n=17, 29.8%). 18 patients (31.0%) had a family history of sudden cardiac death.

28 patients (50.9%) had reported cardiac symptoms prior to their OHCA, including 13 (23.6%) who had experienced syncope. Of the 13 patients who experienced syncope, only three sought medical attention and none had follow-up arranged.

**Conclusions:**

Almost half of paediatric OHCA survivors had warning symptoms prior to their event, representing potential missed opportunities to identify an underlying diagnosis prior to OHCA. These findings highlight the need for more education, both for medical professionals and the general public, about ‘red flag’ symptoms.

**WHAT IS ALREADY KNOWN ON THIS TOPIC**

- Prevention of cardiac arrest and sudden death is a global priority. In line with this, it is important to identify opportunities to diagnose and treat underlying conditions in children believed to be fit and well prior to their presentation with an out-of-hospital cardiac arrest.

**WHAT THIS STUDY ADDS**

- In this retrospective cohort, half of children who had an unexpected out-of-hospital cardiac arrest experienced prior cardiac ‘warning’ symptoms, which may represent a missed opportunity for earlier diagnosis and therefore prevention of cardiac arrest.
- Only a third of all symptomatic patients had contact with a medical professional. These findings support the need for better awareness of cardiac symptoms by both healthcare professionals and local communities.

**HOW THIS STUDY MIGHT AFFECT RESEARCH, PRACTICE OR POLICY**

- Our findings highlight the need for appropriate and timely medical evaluation following cardiac symptoms, especially exertional syncope; there is a need for public health awareness regarding the importance of ‘red flag’ symptoms.
- More work is needed to develop robust strategies to ensure that children and young people who demonstrate cardiac warning symptoms obtain prompt and appropriate evaluation in order to prevent the potentially fatal and devastating consequences of cardiac arrest.

## Background

 Cardiac arrest in children is a rare but devastating event, with an estimated incidence of 9 per 100 000 person years.^1^ Although survival rates have improved in recent years due to progress in resuscitation science and wider availability of defibrillators in the community, less than 10% survive an out-of-hospital cardiac arrest (OHCA, when a cardiac arrest occurs outside of a medical facility) to hospital discharge^2^ and up to 50% of survivors are left with a spectrum of neurological injuries due to hypoxia at the time of arrest.^3^

Approximately half of OHCAs in the young have a cardiac cause.^4^ Cardiac arrest may be the presenting event in children with undiagnosed cardiovascular disease; inherited cardiac conditions (ICCs), including cardiomyopathies and inherited arrhythmia syndromes, are the primary cause of cardiac arrest in the young.^5^ Up to 79% of adult cardiac arrest survivors report previous warning symptoms,^6–8^ but whether the same is true in paediatric OHCA survivors has not been systematically evaluated. Cardiac symptoms, such as syncope and chest pain are relatively common findings^9^ in children and may be benign,^9–12^ but, if not fully investigated, may present a missed opportunity to identify a diagnosis and initiate treatment. This study aimed to describe cardiac symptoms in children prior to their presentation with OHCA.

## Methodology

### Study design and participants

Clinical records from the time of referral and throughout follow-up of consecutive patients aged 0–18 years presenting with a survived OHCA between 1996 and 2025 to a single UK paediatric tertiary referral centre for inherited cardiovascular diseases were retrospectively reviewed. Demographic information, diagnoses and family history were collected. Clinical outcomes included implantable cardioverter-defibrillator (ICD) insertion, appropriate ICD therapy, heart transplant and death.

All patients had documented ventricular fibrillation at the time of OHCA and all patients survived to hospital discharge. Patients were excluded if the cardiac arrest occurred in-hospital, as were patients with a pre-existing condition, including ICCs, deemed to be the cause of their cardiac arrest or where an alternative explanation for the OHCA (eg, non-accidental injury) was identified. Patients in whom the presenting rhythm was not recorded, or where ventricular arrhythmia was present without collapse, were also excluded. Patients with known congenital heart disease (CHD) felt not to be causative of the OHCA, as determined by treating clinicians, were included in the study.

#### Cardiac symptoms

Absence or presence of cardiac symptoms (eg, syncope, presyncope, chest pain, palpitation, shortness of breath or reduced exercise tolerance) at any point prior to the cardiac arrest were recorded where clearly documented, but contemporaneous symptoms at the time of the event were excluded. Cardiac symptoms were assessed by the treating clinicians at the time of presentation from parent and patient reports. If there was no reference to prior symptoms, this was considered missing data. Time from symptom onset to cardiac arrest was recorded if available. Where symptoms were recurrent (eg, two syncopal episodes), the symptom closest to the time of event was used for analysis. Time periods were defined a priori as <1 month, 1–6 months, 6–12 months and >12 months. Non-cardiac symptoms and intercurrent illness at the time of, or just prior to, the event were also recorded, but not included in the analysis of cardiac symptoms.

#### Genetic testing

In patients in whom genetic testing had been performed, the following data were collected: date of testing, size of panel and variant(s) identified. The pathogenicity of reported variants was reclassified by the authors according to American College of Medical Genetics and Genomics criteria.^13^

#### Statistical analysis

All statistical analyses were performed using IBM SPSS V.26.0.0.0. Data are presented as n (%) where categorical; normally distributed continuous variables are described as mean±SD. Skewed data are described as median (IQR). Comparisons were made using the χ² or Fisher’s exact test for categorical variables and Student’s t-test or one-way analysis of variance for continuous variables. A p<0.05 was considered statistically significant for all analyses.

## Results

During the study period, 58 children (female n=29, 50%) presented with OHCA and met the inclusion criteria.

### Demographics and circumstances of cardiac arrest

Table 1 shows the baseline characteristics and circumstances of OHCA. Of 35 patients who arrested in a public setting, 16 (45.7%) were at school and a further 2 (5.7%) collapsed on their way to school. Of 29 patients who arrested on exertion, 11 (37.9%) were playing a team sport at the time of arrest. 12 of 17 patients diagnosed with hypertrophic cardiomyopathy (HCM; 70.5%) presented on exertion, which was the highest proportion of any diagnosis. Two patients (3.4%) experienced cardiac arrest due to a sudden noise (an alarm and a whistle being blown, respectively), one (1.7%) with pain (sustaining an injury) and one (1.7%) with an emotional trigger (an argument).

**Table 1:** Demographic information and circumstances of out-of-hospital cardiac arrest

	Total cohort n=58 (%)	Inherited arrhythmia n=21 (36.2)	Inherited cardiomyopathy n=21 (36.2)	Unascertained n=10 (17.2)	Complex CHD* N=4 (6.8)	P value
Female	29 (50)	12 (57.1)	9 (42.8)	5 (50)	2 (50)	0.836
Age at cardiac arrest (years) Mean±SD	9.96±4.52	7.92±4.77	10.95±3.36	11.45±4.60	11.50±7.04	0.037
<1 year old	4 (6.8)	3 (14.2)	0 (0)	1 (10)	0 (0)	0.3
1–11 years old	33 (56.8)	12 (57.1)	10 (47.6)	3 (30)	1 (25)	0.287
12–18 years old	21 (36.2)	6 (28.5)	11 (52.3)	6 (60)	3 (75)	0.169
Ethnicity						
White	33 (56.9)	11 (52.4)	15 (71.4)	4 (40)	2 (50)	0.358
Asian	9 (15.5)	4 (19.0)	3 (14.3)	0	1 (25)	0.488
African, Caribbean, other black background	8 (13.8)	4 (19.0)	2 (9.5)	2 (20)	0 (0)	0.634
Mixed ethnicity	4 (6.9)	0 (0)	1 (4.8)	3 (30)	0 (0)	0.098
Other ethnic group/not stated	4 (6.9)	0 (0)	0 (0)	1 (10)	1 (25)	0.278
Family history of sudden cardiac death (SCD)	18 (31.0)	6 (28.5)	6 (28.5)	4 (40)	2 (50)	0.774
2nd degree	11 (19)	5 (23.8)	4 (19.0)	0	2 (50)	0.071
3rd degree	7 (12.1)	2 (9.5)	2 (9.5)	3 (30)	0 (0)	0.236
More distant	2 (3.4)	1 (4.7)	0 (0)	1 (10)	0 (0)	0.812
SIDS or sudden death in childhood (<18 years old)	7 (12.1)	3 (14.2)	3 (14.2)	1 (10)	0 (0)	0.789
>1 SCD in the family history	8 (13.8)	5 (23.8)	2 (9.5)	1 (10)	0 (0)	0.566
Place of arrest						
Public place	35 (60.3)	12 (57.1)	15 (71.4)	3 (30)	3 (75)	0.153
At home	19 (32.8)	8 (38.1)	4 (19.0)	6 (60)	1 (10)	0.144
Not stated	4 (6.9)	1 (4.7)	2 (9.5)	1 (10)	0 (0)	
Circumstances of arrest						
On exertion	29 (50)	10 (47.6)	15 (71.4)	1 (10)	2 (50)	**0.016**
At rest	11 (18.9)	2 (9.5)	4 (19.0)	4 (40)	0 (0)	0.016
Sleep	8 (13.7)	6 (28.5)	0 (0)	2 (20)	1 (25)	0.078
Emotional or noise trigger	4 (6.9)	2 (9.5)	0 (0)	1 (10)	0 (0)	0.465
Unwitnessed or not stated	6 (10.34)	0 (0)	0 (0)	0 (0)	0 (0)	

* Where no other cause was found; not believed to be contributory to the OHCA.

CHD, congenital heart disease; SIDS, Sudden Infant Death Syndrome.

Children who arrested during sleep (n=8, 13.7%) had the lowest median age (7.0 years) compared with other circumstances and those who had an arrest on exertion were the oldest (13.0 years) (p=0.007). Three cardiac arrests took place in the context of the patient suffering from an intercurrent febrile illness, one of whom was subsequently diagnosed with Brugada syndrome, one with non-dilated left ventricular cardiomyopathy and one who did not receive a diagnosis.

No patients had a family history of sudden cardiac death (SCD) in a first-degree relative, but 18 patients (31%) had a family history of SCD in a second-degree or more distant relative (table 1). Of these, 16 patients’ relatives (88.8%) had not undergone cardiac screening, and two intervening relatives (11.2%) had undergone cardiac screening with normal findings.

Out of the 42 patients who were diagnosed with ICC, 3 (7.5%) had a known family history of an ICC prior to the OHCA, which had not prompted cardiac screening. These were all family histories of cardiomyopathies, with a diagnosis of HCM being made in each case in the three patients.

Following presentation with OHCA, genetic testing and clinical family evaluation were performed. Of the 38 patients (90.4%) where data were available, 25 patients (65.7%) had a relative diagnosed with an ICC. 17 patients (40.4%) had de novo diagnoses.

Incidence of OHCA across the study period is represented in online supplemental figure S2.

### Diagnoses

Diagnoses are presented in figure 1. Within the patient group diagnosed with ICCs (n=42, 72.4%), 21 patients (50%) were diagnosed with cardiomyopathies and 21 (50%) with inherited arrhythmia syndromes. Two patients were diagnosed with structural, non-inherited cardiac causes for their OHCA (online supplemental table S1): anomalous origin of left coronary artery from non-coronary sinus with intramural course and mitral annular disjunction (n=1, 1.8%, each). Despite thorough phenotypic screening, 14 patients (24.1%) had no cause for the OHCA identified, four of whom (6.8%) also had complex CHD (as per table 1, further information in online supplemental table S2).

**Figure 1:**
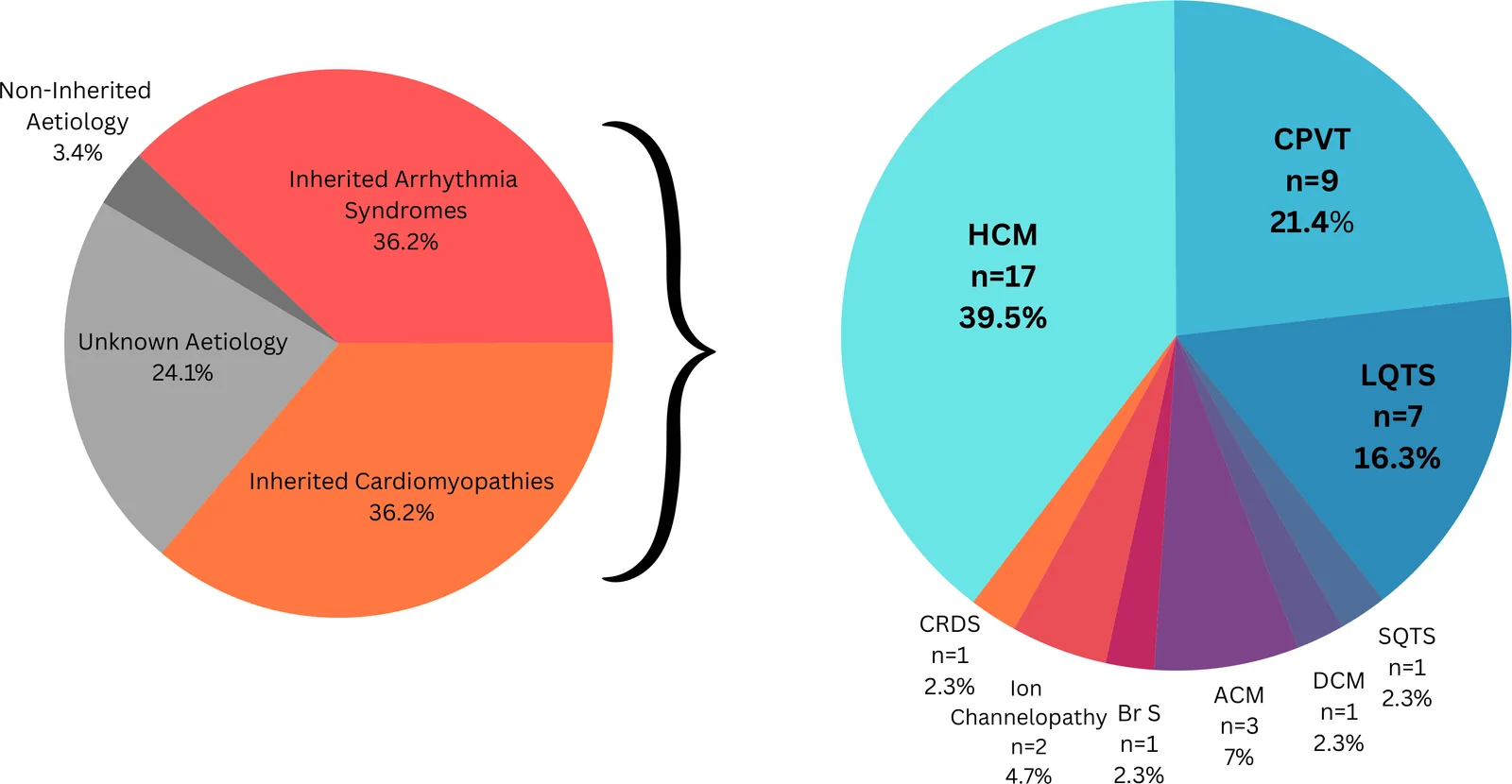
Diagnoses made following out-of-hospital cardiac arrest. ACM, arrhythmogenic cardiomyopathy; BrS, Brugada syndrome; CPVT, catecholaminergic polymorphic ventricular tachycardia; CRDS, calcium release deficiency syndrome; DCM, dilated cardiomyopathy; HCM, hypertrophic cardiomyopathy; LQTS, long QT syndrome; SQTS, short QT syndrome.

### Genetic testing

48 patients (82.8%) underwent genetic testing, of whom 26 (54.1%) had a pathogenic or likely pathogenic (P/LP) variant in an ICC gene identified (online supplemental figure S1 and table S5). Six of these (23.1%) had two P/LP variants identified. 39 out of the 43 patients with a definitive ICC diagnosis (90.6%) underwent genetic testing, with a P/LP variant identified in 26 (66.6%).

### Cardiac symptoms prior to OHCA

28 patients (50.9%) reported cardiac symptoms prior to their presentation with OHCA (figure 2). There were missing data for three patients (5.1%). Syncope was the most commonly reported symptom (n=13, 23.6% of the whole cohort and 46.4% of those with symptoms), followed by chest pain and presyncope (n=6, 10.3%, each). Seven patients (25% of those with symptoms) experienced more than one type of cardiac symptom. In those with syncope; four had more than one episode, and nine (69.2%) had exertional syncope, of whom eight suffered an OHCA on exertion. One patient had both an episode of exertional syncope and an exertional seizure. Of note, six patients were documented to have seizure-like activity during their cardiac arrest.

**Figure 2:**
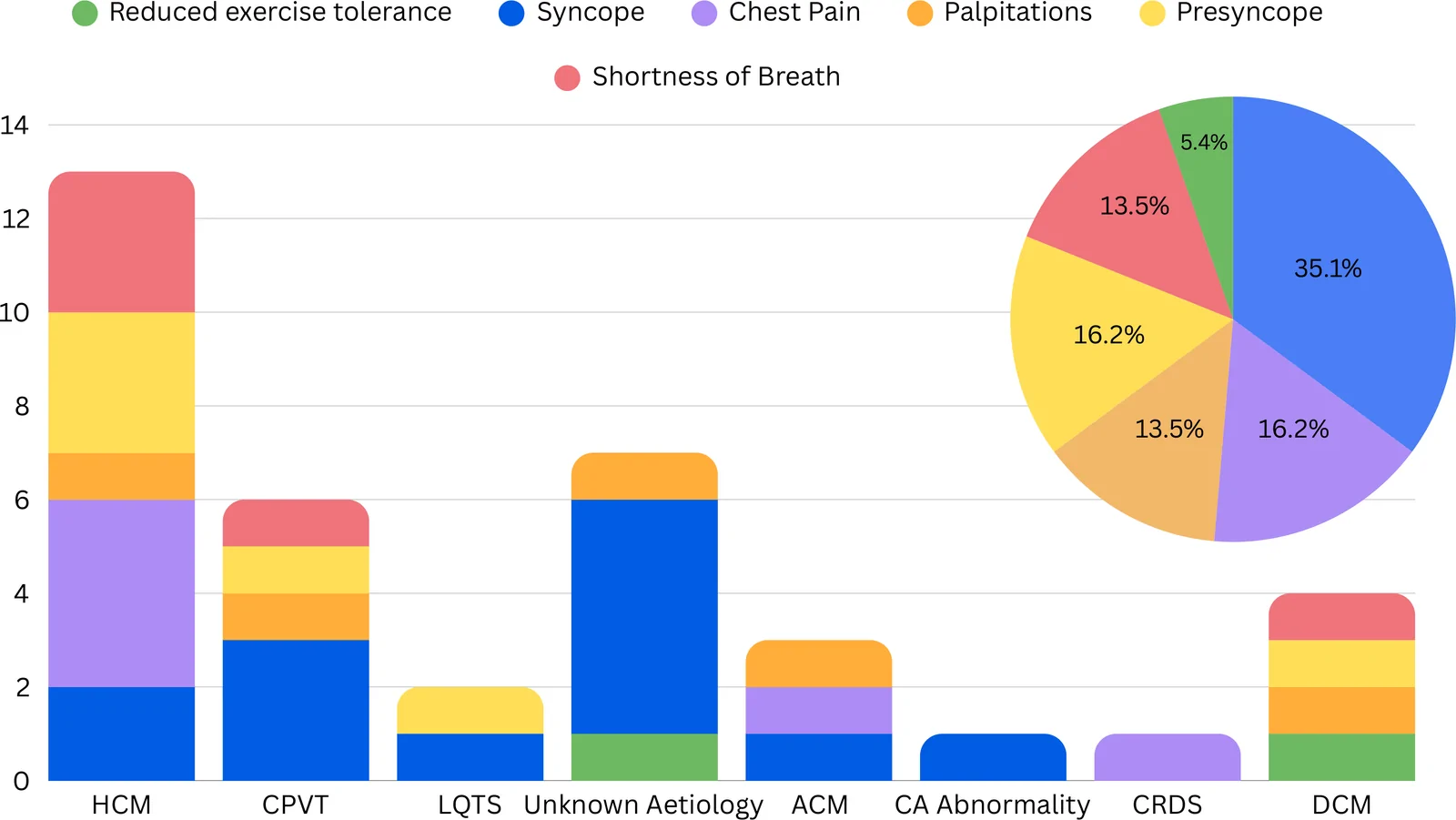
Bar chart showing types of cardiac symptoms reported by diagnosis. Inset–pie chart showing overall symptoms reported by symptom type. ACM, arrhythmogenic cardiomyopathy; CA, coronary artery; CPVT, catecholaminergic polymorphic ventricular tachycardia; CRDS, calcium release deficiency syndrome; DCM, dilated cardiomyopathy; HCM, hypertrophic cardiomyopathy; LQTS, long QT syndrome.

Patients with prior cardiac symptoms were older at the time of their cardiac arrest than those without (11.95 years old±3.02 vs 7.95 years old±5.18, p=0.001), but there were no statistically significant sex differences. Symptoms prior to OHCA were more commonly reported in patients diagnosed with a cardiomyopathy than in those with an inherited arrhythmia syndrome (online supplemental table S4), though this did not reach statistical significance (n=11 (64.7%) vs 9 (40.9%); p=0.058)). Of those who experienced chest pain (n=6), 5 (83%) were diagnosed with a cardiomyopathy and one patient with calcium release deficiency syndrome (CRDS).

Of 25 patients (89.2%) with data available, 5 (15.1%) had symptoms less than 1 month prior to the OHCA, 9 (27.2%) between 1 and 6 months, 9 (27.2%) between 6 and 12 months and 10 (30.3%) >12 months.

Of the 28 patients who had reported any cardiac symptom, nine (32.1%) had been reviewed by a medical professional due to their symptoms before their presentation with cardiac arrest. Of the 13 patients who experienced syncope, only three patients (23.0%, all with exertional syncope) sought medical attention. One was assessed by the ambulance service, one attended the emergency department and one sought advice from their general practitioner. All had a resting ECG performed with no abnormality reported; none had any additional cardiac investigations or further follow-up arranged.

Nine patients (17%) had contact with a cardiologist prior to the cardiac arrest: four patients were under follow-up due to repaired CHD; three were seen due to symptoms (shortness of breath on exertion, palpitations and increased sweating when feeding); one due to neonatal bradycardia and one due to a family history of junctional ectopic tachycardia.

### Congenital defects and structural abnormalities

Four patients (6.8%) had previously repaired CHD (online supplemental table S2), that were deemed not to be the cause of their cardiac arrest. One of these patients, with a repaired ventricular septal defect, was diagnosed with arrhythmogenic right ventricular cardiomyopathy as the cause of the cardiac arrest, with a pathogenic PKP2 variant identified on genetic testing. One patient with repaired transposition of the great arteries was found to have clinical features of an ion channelopathy, but negative genetic testing. The remaining two patients had no cause for their OHCA identified. In all cases, the CHD was not felt to be the cause of the OHCA.

In addition, two patients had CHD or cardiac abnormalities identified on cardiac investigations following arrest, as described in online supplemental table S3. One patient was found to have mitral annular disjunction with mitral valve prolapse, which was suspected to be the cause of their OHCA after extensive investigations had excluded all other potential causes.

### Clinical outcomes

Median follow-up time after OHCA was 4.37 years (IQR 2.25–8 years). Patient outcomes are summarised in table 2. 54 patients (94.7%) underwent secondary prevention ICD implantation prior to hospital discharge. Of those who did not receive an ICD, one had surgical repair of an anomalous origin of left coronary artery from non-coronary sinus and three had poor neurological prognoses and ICD implantation was declined as part of a shared decision-making process. 11 patients (18.9%) suffered significant neurological sequelae due to the cardiac arrest.

**Table 2:** Clinical outcomes

	Total cohort=58 n (%)
Length of follow-up, median (years)	4.37 (IQR 2.25–8)
ICD implanted n (%)	54 (94.7)
Appropriate ICD therapy	17 (31.4)
Cardiac transplant n (%)	2 (3.5)
Death n (%)	6 (10.3)
Good neurological outcome	42 (72.4)
Neurological injury	11 (18.9)
Not stated	5 (8.6)

ICD, implantable cardioverter-defibrillator.

During follow-up, two patients diagnosed with catecholaminergic polymorphic ventricular tachycardia had left cardiac sympathetic denervation performed (in addition to ICD implantation) and two patients (3.5%) underwent cardiac transplantation (diagnosis of HCM (n=1) and dilated cardiomyopathy (DCM) (n=1)), 7 years and 9 months after OHCA, respectively). Six patients (10.3%) died during follow-up: four in childhood and two following transition to adult services. Median age of death was 16.5 years old (IQR=12–200.25). Causes of death were recorded as: intractable arrhythmia (n=1), transplant rejection (n=1), sudden death with non-shockable rhythm (n=1) and not stated (n=3).

## Discussion

This study demonstrates that half of child-age patients presenting with OHCA had experienced cardiac symptoms prior to their cardiac arrest, representing potentially missed opportunities for prevention.

### Identification of cardiac symptoms in children

Potential cardiac symptoms are common and often non-specific in childhood, which can make it challenging for clinicians to identify those individuals with a cardiac cause for their symptoms. Syncope is particularly common in the paediatric population and often vasovagal in nature, with the most common age for first onset of vasovagal syncope at 13 years of age.^14^ However, exertional syncope is recognised as a ‘red flag’ symptom and is highly suspicious for cardiogenic syncope.^15 16^ Following a syncopal event, patients may come under the care of a range of specialities and the lack of a clear pathway has the potential to contribute to misdiagnosis and inappropriate treatment. Similarly, chest pain is often non-cardiac in children without a known cardiovascular disorder and it can be difficult to differentiate this from other more common causes, such as musculoskeletal pain, including costochondritis,^17^ gastrointestinal pain or respiratory chest pain associated with asthma.

The reporting of symptoms and their interpretation can pose additional challenges within paediatric care. This includes non-specific presentation in children, age-related communication barriers or applicability of established, often adult-focused, diagnostic tools.^18^ In this study, patients with prior cardiac symptoms were older at the time of their cardiac arrest than those without (11.95 years old±3.02 vs 7.95 years old±5.18, p=0.001), which may be due to older children being more likely to recall or accurately report them. In addition, the manifestation of symptoms in younger children can be different to those more typically described in older children and adolescents.

Patients in this study had varying interactions with a range of healthcare professionals prior to their presentation with OHCA. Some patients had incidental contact with healthcare professionals, including cardiologists, for reasons unrelated to symptoms they had experienced, including cardiac diagnoses not felt to be the cause of the eventual cardiac arrest. Importantly, most patients who experienced syncope did not seek medical advice after their syncopal event. Factors affecting patients and families contacting healthcare services following a syncopal episode may include an attitude that syncope is ‘not serious’, or they are overburdening the system.^19^ A Danish study reported that 35% of individuals with a sudden arrhythmic death syndrome (SADS) postmortem had cardiac symptoms prior to death but less than a quarter had reported them to a healthcare professional.^20^

### Exertional symptoms prior to cardiac arrest

Our data are in keeping with previous studies describing a relatively high number of patients reporting symptoms prior to OHCA,^21 22^ although previous study designs included individuals who were not successfully resuscitated and used retrospective questionnaires^21^ or next of kin, pathologist and coroner reports to collate evidence of premortem cardiac symptoms.^22^ Therefore, these data may be limited by recall bias and parental or caregiver reports.

While OHCA during exercise is reported to occur in approximately 10% of cases in largely adult studies,^4 23^ a much higher proportion (50%) was observed in the present study. We speculate that this could be due to children spending a larger proportion of their time being more physically active than adult counterparts. It is also possible that this represents a survival bias, as perhaps children who are more active are generally healthier and may therefore be more likely to survive. Additionally, the underlying aetiology of children who present with OHCA may differ from the adult population.^24^

Of note, one patient in this study had an episode described as an exertional seizure prior to OHCA. Ventricular arrhythmia presenting as a seizure may be confused with a neurological presentation and delay resuscitation, but exertion triggered seizure-activity should be considered a ‘red flag’ symptom. In keeping with this, six patients were described as having a seizure during their cardiac arrest. It is likely that this is an underestimation as the presentation of all cardiac arrests was not described fully in the clinical records.

Overall, our results highlight the need for increased awareness of the importance of cardiac symptoms, in particular exertional syncope and full investigation of these, in line with national and international guidelines.^15 25^

### Yield of ICCs in childhood OHCA survivors

This study demonstrates a high yield (72.4%) of ICCs diagnosed in patients presenting with OHCA when referred to a specialist service. Limited data exist on the causes of successfully resuscitated OHCA in children,^1^ and most available literature reporting cause of cardiac arrest or SCD in the young relies on postmortem data and subsequent family screening.^5 23^ These postmortem data demonstrate a high proportion of autopsy-negative, or SADS SCDs, indicating possible inherited arrhythmia syndromes.

Following cardiac evaluation, 12.3% of adult-age cases of sudden cardiac arrest (SCA) will remain unexplained.^26^ This is lower than our cohort (24.1%), which could be accounted for by the era in which investigations were performed in some patients and incomplete and age-related penetrance of some cardiac conditions in children.^27^

Nearly one-third of the cohort had a family history of SCD in a second degree or more distant relative. There was no screening of the intervening relatives in the majority of cases, suggesting further potential missed opportunities for earlier detection. In addition, two-thirds of patients were found to have familial inherited cardiovascular disease following their cardiac arrest, which may be associated with missed opportunities in relation to family screening prior to their OHCA.

This study included four patients with previously known but repaired CHD. Although SCD is well-described in this population, the mechanism is not always clear.^28^ ICC diagnoses were made in two children with CHD, highlighting that these conditions can co-exist and the importance of searching for a cause that can explain the cardiac arrest.

### Prevention of SCA in the young: education and awareness

Prevention of SCD has been recognised as a global priority.^29^ Several solutions have been suggested, including population screening, preparticipation athletic screening and establishing paediatric syncope clinics. Preventing OHCA in children not only aims to prevent SCD, but in the survivors, potentially devastating neurological and psychological outcomes. Cardiac symptoms in children represent a potential opportunity to identify an underlying cause of OHCA and therefore prevent an event with wide-ranging consequences for the young person, their family and wider community.

Near-term prevention and pre-emptive action have recently been highlighted^30^ as important strategies in efforts to address SCD, which act to bridge the gap between long-term prevention approaches and emergency resuscitation. Identifying those individuals at risk of ventricular arrhythmia and offering rapid evaluation is key. Potential future tools such as artificial intelligence being employed to provide risk assessment for ambulatory ECGs may help clinicians risk stratify symptomatic patients.^31^

Previous studies have demonstrated that recognition of cardiac symptoms, especially in the 4 weeks prior to a SCA, is associated with increased survival.^32^ Although the reason for this is unclear, this also highlights the importance of treating symptoms as an opportunity for intervention.

A high proportion of both symptoms and OHCA occurred at school or sports clubs. Provision of automatic external defibrillators in public spaces is a well-recognised strategy to prevent SCD, and is associated with a near doubling of 30-day survival^33^ and increased neurologically-favourable survival in children.^34^ The limited number of families who sought medical attention for exertional symptoms in this study, together with the fact that a large number of events occurred at school or sports clubs, suggests that more education should be directed towards sports coaches and teachers. In particular, an increased awareness of the importance of medical attention following exertional syncope or ‘seizure-like’ episode could be an important goal of a prevention strategy.

## Limitations

This study is limited by missing or incomplete clinical data, inherent to its retrospective nature and not all patients were cared for in line with current guidelines. This means that not all diagnostic tests were performed in every patient, and some children presented before the widespread availability of genetic testing. It is therefore possible that the true overall yield of ICC diagnoses is higher than stated in this study. Symptom reporting was based on contemporaneous history taking from patients and families by specialist medical or nursing staff, which should reduce the effect of recall bias. However, in common with other similar studies, it is still possible that symptom reporting was affected by incomplete documentation or attentional bias. In addition, in common with other studies investigating prior symptoms often employing a retrospective design in which patients and/or family members complete a survey or questionnaire after discharge, there may be recall bias in the reporting of symptoms. However, all notes were from specialist medical or nursing staff, taken at the time of or shortly after clinical presentation, making this less likely.

## Conclusions

Almost half of paediatric-age OHCA survivors had cardiac warning symptoms prior to their event, including over 20% with previous syncope. These represent potential missed opportunities to identify an underlying diagnosis and implement preventive treatment prior to a cardiac arrest. The findings from this study highlight the need for more education, both for medical professionals and the general public, about ‘red flag’ cardiac symptoms.

## Supplementary material

10.1136/openhrt-2026-004412 online supplemental file 1

## Data Availability

Data are available on reasonable request.
