# Supplementary material for: Cardiac warning symptoms in paediatric out-of-hospital ventricular fibrillation cardiac arrest survivors
Source: Open Heart. 2026 Oct 5;13(2):e004412. doi: 10.1136/openhrt-2026-004412 (PMC13647030; doi:10.1136/openhrt-2026-004412)
Supplement: Supplementary file 1 [file openhrt-13-2-s001.docx]

**Supplementary Material**

**Table S1. Diagnoses in cohort**

| **Diagnosis made following OOHCA** | Total cohort = 58 *n*(%) |
| --- | --- |
| **Inherited Cardiac Condition**  Hypertrophic Cardiomyopathy  CPVT  Long QT Syndrome  Arrhythmogenic Cardiomyopathies  *Non-Dilated LV Cardiomyopathy*  *ARVC*  Ion Channel Disease  CRDS  Brugada Syndrome  Short QT Syndrome  DCM    **Unknown Aetiology**  **Non-inherited cause**  *Anomalous origin of left coronary artery from non-coronary sinus with intramural course*  *Mitral valve prolapse with mitral annular disjunction* | 42 (72.4)  17 (29.8)  9 (15.8)  7 (12.3)  3 (5.3)  2 (3.5)  1 (1.8)  2 (3.5)  1 (1.8)  1 (1.8)  1 (1.8)  1 (1.8)    14 (24.1)     2 (3.4)  1 (1.8)  1 (1.8) |

**Table S2 Congenital heart defects and structural abnormalities diagnosed prior to arrest**

| **CHD Diagnosis/Cardiac Finding** | **Sex** | **Timing of diagnosis** | **Cause of OHCA** | **Age at OHCA** | **Surgery prior to OHCA** | **Details** |
| --- | --- | --- | --- | --- | --- | --- |
| Transposition of the Great Arteries (TGA), Ventricular Septal Defects (VSDs) | Female | Neonate | Suspected ion channelopathy | 16 months old | Arterial switch with VSD closure | Last clinical review 5 months prior to OHCA demonstrated good systolic function, no outflow tract obstruction and small residual VSD.  Coronary angiogram performed following OHCA demonstrated unobstructed coronary arteries. |
| TGA, VSD and pulmonary stenosis | Female | Neonate | Unascertained | 16 years old | Balloon atrial septostomy; Right modified BT shunt Rastelli procedure; Percutaneous pulmonary valve implantation x2 | Last clinical review six months prior to OHCA demonstrated mild to moderate right ventricular hypertrophy , good systolic function outflow tract obstruction? |
| Small peri-membranous VSD | Female | Neonate | ARVC secondary to pathogenic PKP2 variant | 14 years old | VSD repair | Last clinical review two years prior to OHCA. Normal systolic function and no residual VSD at last echocardiogram nine years prior to OHCA. |
| Truncus Arteriosus Type 1 | Male | Three months old | Unascertained | 15 years old | Truncus arteriosus repair; Redo RV to PA conduit with homograft; Percutaneous pulmonary valve implantation x 2 | Last clinical review seven months prior to OHCA - mildly dilated and hypertrophied right ventricle and normal systolic function |

**Table S3 Congenital heart defects and structural abnormalities found after cardiac arrest which were not deemed to be the cause of the cardiac arrest**

| **CHD Diagnosis/Cardiac Finding** | **Sex** | **Cause of OHCA** | **Age at OHCA** | **Details** |
| --- | --- | --- | --- | --- |
| Subaortic membrane and single coronary artery rising from the left coronary sinus | Male | Unascertained | 14 years old | Following OOHCA echocardiogram demonstrated bicuspid aortic valve with subaortic membrane,  Vmax AV of 3.8msec, mild aortic regurgitation and mild-moderate concentric left ventricular hypertrophy. Single left coronary orifice detected on coronary angiogram. |
| Dysplastic aortic valve with moderate aortic arch dilatation | Male | CPVT | 10 years old | Following OHCA echocardiogram and CMR demonstrated moderate aortic root dilatation, 40mm at right non-coronary sinus and mild dilatation of proximal descending aorta with mild aortic regurgitation and no left ventricular hypertrophy. No aortopathy diagnosed; genetic testing for aortopathy negative. |

**Table S4 Symptoms by underlying aetiology of OHCA**

| **Symptom** | **Total Cohort *n* = 55 (%)** | **Inherited Arrhythmia**  ***n*=21 (38.1)** | **Inherited Cardiomyopathy**  ***n*=19 (34.5)** | **Unascertained**  ***n*=13**  **(23.6)** | ***p value*** |
| --- | --- | --- | --- | --- | --- |
| **Syncope** | 13 (23.6) | 4 (19.0) | 3 (15.7) | 5 (38.4) | .316 |
| **Presyncope** | 6 (10.9) | 2 (9.5) | 4 (21.0) | 0 (0) | .372 |
| **Chest pain** | 6 (10.9) | 1 (4.7) | 5 (26.3) | 0 (0) | .112 |
| **Palpitations** | 5 (9.0) | 1 (4.7) | 3 (15.7) | 1 (7.7) | .690 |
| **Reduced exercise tolerance** | 2 (3.6) | 0 (0) | 1 (5.2) | 1 (7.7) | .733 |
| **Shortness of breath** | 4 (7.2) | 0 (0) | 4 (21.0) | 0 (0) | .094 |

**Table S5 Genes in which pathogenic and likely pathogenic variants were identified**

| **Diagnosis** | **Gene** |  | **Patients (n)** |
| --- | --- | --- | --- |
| **HCM** | *MYBPC3* | Heterozygous | 3 |
|  |  | Compound Heterozygous | 3 |
|  | *TNNT2* | Heterozygous | 2 |
|  | *TNNI3* | Heterozygous | 2 |
|  | *MT-TL1/TNNI3* | Heterozygous | 1 |
|  | *MYH7* | Heterozygous | 1 |
| **CPVT** | *RYR2* | Heterozygous | 5 |
|  | *TECRL* | Compound Heterozygous | 1 |
|  | *TRDN* | Compound Heterozygous | 1 |
| **LQTS** | *CACNA1C* | Heterozygous | 1 |
|  | *SCN5A* | Heterozygous | 1 |
| **ACM** |  |  |  |
| NDLV | *DSG2* | Heterozygous | 1 |
| ARVC | *PKP2* | Heterozygous | 1 |
| **Brugada Syndrome** | *SCN5A* | Heterozygous | 1 |
| **SQTS** | *SLC22A5* | Homozygous | 1 |

**Figure S1 Genes in which pathogenic and likely pathogenic variants were identified**

|  | Number |
| --- | --- |
| 1996-2001 | 2 |
| 2002-2007 | 6 |
| 2008-2013 | 16 |
| 2014-2019 | 17 |
| 2020-2025 | 17 |

**Figure S2 Incidence of OHCA across study period by 5-yearly strata**
